# Supplementary material for: Dehumanization and mass violence: A study of mental state language in Nazi propaganda (1927–1945)
Source: PLoS One. 2022 Nov 9;17(11):e0274957. doi: 10.1371/journal.pone.0274957 (PMC9645591; doi:10.1371/journal.pone.0274957)
Supplement: S1 File — (DOCX) [file pone.0274957.s001.docx]

**Supporting Information**

**Additional linguistic constructs: detailed results**

We conducted additional interrupted time series analyses to examine the temporal dynamics of five other relevant linguistic categories in the antisemitic Nazi propaganda: negative emotions, affiliation, health, purity, and death. As with our other time series analyses, we checked for autocorrelation with Box-Ljung tests. There was no autocorrelation in the affiliation or health data, but there was significant autocorrelation in the negative emotions, purity, and death data. For these latter three datasets, we evaluated the base models relative to autoregressive AR(1) models by comparing their corrected Akaike information criteria (AICc). In each case, the base model was a better fit to the data. Moreover, Ljung-Box tests on the residuals from these fitted models indicated they accounted for all autocorrelation. Nonetheless, the same patterns of results emerged when using the AR(1) models instead of the base models.

We analyzed the prevalence of *negative emotions* to evaluate the possibility that the decline in experience preceding the Holocaust in the Jew-relevant text was driven by general negative sentiment. We found no evidence for this possibility, as there was no trend in negative emotions preceding the Holocaust, *b*_1_ = 0.004, 95% CI [-0.01, 0.01], *SE* = 0.005, *t* = 0.72, *p* = .47. There was a marginal increase in the level of negative emotion at the onset of the Holocaust, *b*_2_ = 1.41, 95% CI [-0.01, 2.83], *SE* = 0.71, *t* = 1.98, *p* = .052, and no change in the trend from pre- to post-onset, *b*_3_ = 0.02, 95% CI [-0.02, 0.06], *SE* = 0.02, *t* = 1.07, *p* = .29. We also sought convergent support for our finding that experience terms decreased preceding the Holocaust by analyzing *affiliation* terms (e.g., ally, friend), because a desire for affiliation can motivate people to recognize other’s mental experiences [1]. Consistent with the declining trend in experience, we observed a marginally significant decline in affiliation prior to the onset of the Holocaust, *b*_1_ = -0.01, 95% CI[-0.02, 0], *SE* = 0.004, *t* = -1.83, *p* = .074. There was no change in the level of affiliation at the onset of the Holocaust, *b*_2_ = 0.20, 95% CI [-0.97, 1.36], *SE* = 0.58, *t* = 0.34, *p* = .74, and no change in the trend from pre- to post-onset, *b*_3_ = -0.01, 95% CI [-0.05, 0.02], *SE* = 0.02, *t* = -0.78, *p* = .44.

Conceptual metaphors equating Jews to diseases infecting Germany’s “national body” (*Volkskörper*) were central to Nazi antisemitism [2,3], contributing to their dehumanization and possibly motivating the desire to completely exterminate them [4,5]. Indeed, eminent Nazis promised to cleanse the world of a purported “Jewish world plague” [5]. Therefore, we analyzed terms related to *health* (e.g., clinic, flu) and *purity* (e.g., pious, pristine). In line with the notion that conceptual metaphors of disease and a desire for purity motivated mass violence against the Jews, we observed a significantly increasing trend in health terms in the lead up to the Holocaust, *b*_1_ = 0.01, 95% CI [0.003, 0.01], *SE* = 0.002, *t* = 3.66, *p* < .001. We also observed a significant positive trend in purity terms preceding the Holocaust, *b*_1_ = 0.002, 95% CI [0.001, 0.003], *SE* = 0.0004, *t* = 5.40, *p* < .001. There was no change in the level of health terms at the onset of the Holocaust, *b*_2_ = -0.02, 95% CI [-0.44, 0.41], *SE* = 0.21, *t* = -0.07, *p* = .94, and a significant negative change in the trend from pre- to post-onset, *b*_3_ = -0.02, 95% CI [-0.03, -0.01], *SE* = 0.01, *t* = -2.89, *p* = .006. There was a marginal increase in the level of purity terms following the onset of the Holocaust, *b*_2_ = 0.11, 95% CI [-0.01, 0.22], *SE* = 0.06, *t* = 1.82, *p* = .074, and a significantly negative change in slope from pre- to post-onset, *b*_3_ = -0.004, 95% CI [-0.01, -0.001], *SE* = 0.002, *t* = -2.83, *p* = .007. We also observed an increase in terms related to *death* (e.g., kill, coffin) leading up to the onset of the Holocaust, *b*_1_ = 0.01, 95% CI [0.01, 0.02], *SE* = 0.002, *t* = 6.91, *p* < .001. Death terms also showed an increase in level at the onset of the Holocaust, *b*_2_ = 0.54, 95% CI [0.01, 1.08], *SE* = 0.27, *t* = 2.03, *p* = .048, and a negative change in trend from pre- to post-onset, *b*_3_ = -0.02, 95% CI [-0.04, -0.01], *SE* = 0.01, *t* = -2.74, *p* = .008.

Another factor thought to motivate the Nazis’ escalation of antisemitic policies to genocide was the increasing sense of threat Jews were perceived to pose after their ill-fated invasion of the Soviet Union in late June, 1941—which roughly coincided with the beginning of the Holocaust in July of that year [6,7]. To evaluate this possibility, we performed a two-sample t-test with 1000 resamples to compare the mean proportion of threat terms in the Nazi propaganda published before the Holocaust and that published after its onset. Indeed, threat terms were significantly higher in the propaganda published after the onset of the Holocaust than before, *M*s = 2.05 vs 3.81, *d* = 1.31, *t*(56) = -4.97, *p* < .001, 95% CI [-2.57, -1.08], supporting the possibility that threat increased following the onset of the Holocaust.

**Supporting information references**

1 Waytz, A. and Young, L. (2014) Two motivations for two dimensions of mind. *Journal of Experimental Social Psychology* 55, 278–283

2 Musolff, A. (2007) What role do metaphors play in racial prejudice? The function of antisemitic imagery in Hitler’s *Mein Kampf*. *Patterns of Prejudice* 41, 21–43

3 Snyder, T. (2015) *Black Earth: The Holocaust as History and Warning*, Tim Duggan Books.

4 Neilsen, R. and University of Queensland (2015) “Toxification” as a More Precise Early Warning Sign for Genocide Than Dehumanization? An Emerging Research Agenda. *GSP* 9, 83–95

5 Savage, R. (2007) “Disease Incarnate”: Biopolitical Discourse and Genocidal Dehumanisation in the Age of Modernity. *Journal of Historical Sociology* 20, 404–440

6 Rees, L. (2017) *The Holocaust: A New History*, PublicAffairs.

7 Hayes, P. (2017) *Why?: Explaining the Holocaust*, W. W. Norton & Company.
