# Supplementary material for: Dehumanization and mass violence: A study of mental state language in Nazi propaganda (1927–1945)
Source: PLoS One. 2022 Nov 9;17(11):e0274957. doi: 10.1371/journal.pone.0274957 (PMC9645591; doi:10.1371/journal.pone.0274957)
Supplement: S1 Table — a Internal sources were those produced for Nazi party members (e.g., bulletins instructing Nazi leaders on how to compose their speeches). b External sources were those produced for a public audience (e.g., widely disseminated posters). (DOCX) [file pone.0274957.s002.docx]

**S1 Table. Information on the propaganda comprising our corpus.**

| **Source Type** | **# of Sources** | **Average Word Count** | **# of Internal Sources**^a^ | **# of External Sources**^b^ |
| --- | --- | --- | --- | --- |
| Poster | 12 | 37.17 | 0 | 12 |
| Newspaper | 10 | 1071.70 | 2 | 8 |
| Book | 3 | 375.00 | 0 | 3 |
| Magazine | 5 | 48.20 | 0 | 5 |
| Speech | 17 | 351.29 | 0 | 17 |
| Essay | 1 | 51.00 | 0 | 1 |
| Pamphlet | 7 | 994.86 | 0 | 7 |
| Article | 35 | 471.74 | 3 | 32 |
| Letter | 2 | 506.00 | 0 | 2 |
| Bulletin | 34 | 297.38 | 30 | 4 |
| Newsletter | 14 | 171.71 | 11 | 3 |
| Total | 140 | 396.81 | 46 | 94 |

^a^ Internal sources were those produced for Nazi party members (e.g., bulletins instructing Nazi leaders on how to compose their speeches)

^b^ External sources were those produced for a public audience (e.g., widely disseminated posters)
