# Supplementary material for: Dehumanization and mass violence: A study of mental state language in Nazi propaganda (1927–1945)
Source: PLoS One. 2022 Nov 9;17(11):e0274957. doi: 10.1371/journal.pone.0274957 (PMC9645591; doi:10.1371/journal.pone.0274957)
Supplement: Attachment — Submitted filename: Response to Reviewers.docx [file pone.0274957.s003.docx]

July 27, 2022

To whom it may concern:

My colleagues and I would first like to sincerely thank the editor and four anonymous reviewers for their careful consideration of the initial draft our manuscript, entitled “Dehumanization and mass violence: A study of mental state language in Nazi propaganda (1927-1945)” (Manuscript ID: PONE-D-21-38261). We respectfully request your reconsideration.

We received gracious feedback from the reviewers on July 20, 2022. In total, they offered 14 distinct actionable suggestions to improve our manuscript. We took each piece of advice into careful consideration, and I hope this is reflected in an improved manuscript. In the following, I will respond to each distinct point raised by the reviewers, noting our revisions, in the order they appeared in the original decision letter.

Firstly, we note that Reviewers 1 and 2 each made several general suggestions that we believe we address in our revisions based on the more targeted suggestions of Reviewers 3 and 4. Namely, Reviewer 1 asked us to “improve the presentation and discussion of the results”, while Reviewer 2 suggested we discuss the procedure for data collection, methodology, and results in more detail.

**Contemporary relevance of our research.** Given that our study was focused on a specific historical context (Nazi propaganda published from 1927-1945) Reviewer #2 asked us to elaborate on the relevance of our work today. We first note that throughout the introduction and conclusion, we place theoretical emphasis on a current tension in the dehumanization literature of interest to many contemporary social psychologists—that is, differing perspectives on dehumanization’s role in mass violence. As discussed in the main text (see especially the subsection of the introduction titled “Mind denial and mass violence: moral disengagement or limited relevance?”; *lines 56-87*), it was long assumed that dehumanization facilitates violence by disengaging moral restraints against harming conspecifics (e.g., [Kelman, 1973](https://doi.org/10.1111/j.1540-4560.1973.tb00102.x)). However, recent prominent critiques have questioned this hypothesis on both conceptual and empirical grounds (e.g., [Bloom, 2017](https://www.newyorker.com/magazine/2017/11/27/the-root-of-all-cruelty); [Enock, 2022](https://doi.org/10.1016/j.cognition.2021.104865); [Lang, 2020](https://doi.org/10.1016/j.copsyc.2020.02.002); [Over, 2021](https://doi.org/10.1177/1745691620902133)). Our study offers an ecologically valid test of these competing perspectives that we believe to be of interest for contemporary social psychologists, and say as much in our Discussion (*lines 566-580*).

Moreover, we discuss how this work has implications for future research. Namely, we suggest that our study lays a foundation for further empirical scrutiny of dehumanization—and other social-psychological constructs—in ecologically valid contexts, particularly by leveraging recent advances in computational social science (e.g., [Mendelsohn et al., 2020](https://doi.org/10.3389/frai.2020.00055); *see Abstract and lines 708-711, 718-722*).

**Revised Figure 2**. Reviewer #2 noted the low quality of our figures. In light of this, we revised Figure 2. To more clearly show the dispersion of data and trend line, we shortened the Y-axis and made the datapoints and smoothed regression lines smaller. We also altered the color palette in an effort to make the figure more aesthetically pleasing. We believe Figures 1 and 3 are of sufficient quality and therefore did not revise them.

**Describing the German Propaganda Achieve.** Reviewer 3 asked us to provide more deail on our source of Nazi propaganda, so we now expand on our description of the German Propaganda Achieve: “Established in 1999, this voluminous archive now contains hundreds of Nazi-era posters, articles, pamphlets, books, newspapers, and transcripts of political speeches (see “Examples of Nazi propaganda” in Supporting Information). These materials were intended for	a variety of audiences, including children, the general public, and members of the Nazi party. The original German-language versions of the propaganda were collected from the German Federal Archives, university libraries, and the archivist’s personal collection used for his own research (see <https://research.calvin.edu/german-propaganda-archive/faq.htm> for more information on the archive)” (*lines 191-199*).

**Elaborating on data extraction**. Reviewers 1 and 3 each asked us to provide more details on how the control text was extracted from the propaganda. We have now provided additional detail on the development of the control text: “After extracting all text referring to Jews to form the primary dataset (hereby ‘antisemitic text’), the third author returned to each piece of propaganda that was included in this dataset and extracted the same number of words *not* already coded as referring to Jews to form a control text. The exception was cases where over half of the text was already extracted because it referred to Jews (*n* = 17). In these instances, we simply took all of the remaining text from that piece of propaganda” (*lines 239-244*).

**Justifying our level of analysis.** Reviewer 3 asked why we chose the month as our level of analysis (rather than, for instance, dividing the data into bimonthly or seasonal components). We decided to analyze the data at the level of the month in order to be as inclusive as possible without unduly compromising internal validity. This was included in our preregistered analysis plan: <https://aspredicted.org/GZC_7X1>. Nonetheless, we readily accept that there are alternative ways of analyzing these data and provide both the raw data and analysis script on an open-access repository should others wish to perform these alternative analyses: <https://osf.io/n2kym/?view_only=514019c7ac3d41f39e8fb35616fb688a>

**Keyness plot**. Reviewer 3 noted that the methodology and results from the Keyness plots we constructed could have been elaborated on. We do so now in the “Keyness plots of agency and experience in antisemitic text” subsection of the Results (*lines 455-492*). For one, we elaborate on the methodology used to construct the plots and provide supporting references for a more comprehensive overview: “These plots were derived using Chi-square tests that compared the relative frequency of a word in one subset of the text against the expected frequency based on the other subset (e.g., the experience term “hatred” appeared much more frequently in the post-onset subset of text than would be expected given its frequency in the pre-onset text, while “honor” was much less so; [Scott, 1997](https://doi.org/10.1016/S0346-251X(97)00011-0); [Scott & Tribble, 2006](https://benjamins.com/catalog/scl.22))” (*lines 457-461*). We also expand on the results in this section, “Examining agency terms (Fig 3a), those referring to intentionality (e.g., plans, goal) and malevolence (infernal, evil) increased following the onset of the Holocaust, while those referring to benevolence (love, benevolent) decreased. Likewise, several malevolent experience terms (e.g., sadistic, hatred) increased following the onset of the Holocaust, while some benevolent experience terms (honor, concerned) decreased (Fig 3b)” (*lines 462-491*). We elaborate on these results in the discussion when speculating that the Jews were subject to demonization following the onset of the Holocaust: “after the onset of the Holocaust, we observed an *increase* in agentic mental state terms—specifically terms related to intentionality and malevolence (Fig 3a). Several experience terms referring to malevolence also increased following the onset of the Holocaust, as did the term “guilty”—with its clear implication of moral culpability (Fig 3b). These patterns are consistent with claims that the Jews were subject to *demonization*, in which their capacity for sophisticated reasoning was perceived to coexist with a subhuman moral depravity [2,4] (see also [53,54])” (*lines 580-600*).

**Agency and experience correlation.** In the Introduction of our original manuscript, we support the claim that “Agency and experience are distinct dimensions of mind but are often moderately correlated” by presenting the correlation (*r* = .32) from our own data. Reviewer 4 suggested we instead move this empirical result to the Results section and support the claim with another reference. We have done so, supporting our assertion by referencing seminal papers on mind perception ([Waytz et al., 2010](https://doi.org/10.1016/j.tics.2010.05.006); [Gray et al., 2007](https://www.science.org/doi/full/10.1126/science.1134475?casa_token=TKEGuMQ80-YAAAAA%3AGgPUxDDefZS7KSlkqdLYOv8UFtXrPBVBrl8_3tKI1lz6_1Jf8uxJ9UA2_919qc3tcPKoU6CiIuvCs8tW); *line 66*) and moved the reporting of the correlation between the proportion of agency and experience terms in our own data to the Results section—including the accompanying *N* and confidence interval (*lines 348-349*).

**Restructuring our presentation of the additional linguistic constructs.** Along with our focal construct of mental state terms, we also analyzed several additional linguistic constructs that were theoretically relevant to either dehumanization or the historical context in which we were studying it—the Nazi regime. However, Reviewers 3 and 4 both noted that the introduction, operationalization, reporting of results, and discussion of these additional analyses and constructs was disorganized and difficult to follow. Upon further review, we concur and have thus substantially reorganized the presentation of these constructs.

In the introduction, we now clearly introduce each of these additional constructs in a subsection titled “Additional linguistic constructs” (*lines 124-155*). This subsection provides the theoretical rationale for investigating each of the six constructs we examined, on the basis of critiques and theories of dehumanization (for negative emotion and affiliation terms; *lines 127-133*) or historical accounts of the Nazi regime (for health, purity, death, and threat terms; *lines 134-155*).

We clarify the operationalization of these additional constructs in the subsection of the Method section titled “Psycholinguistic tools for additional linguistic constructs” (*lines 247-271*), describing the psycholinguistic tools used to operationalize negative emotion, affiliation, health, and death terms (the default LIWC2015 dictionary; [Pennebaker et al., 2015](http://hdl.handle.net/2152/31333)), purity terms (the Moral Foundations Dictionary for LIWC; [Graham et al., 2009](https://doi.org/10.1037/a0015141)) and threat terms (the Threat Dictionary; [Choi et al., 2022](https://doi.org/10.1073/pnas.2113891119)). We also provide an overview of the analytic procedure for each of these linguistic constructs in the “Additional analyses” subsection of the Analysis Plan (*lines 317-331*).

We provide a narrative overview of the results of these analyses in the main text (*lines 495-565*). As in the introduction, we structure this section by organizing the results for each construct in terms of the theoretical rational for analyzing it (i.e., results related to dehumanization theory and those related to factors thought to contribute to the Holocaust. Along with this narrative overview, we provide detailed reporting of the results in the Supporting Information (*lines 2-56*).

Finally, we have created a new subsection in the Discussion titled “Additional linguistic constructs” (*lines 662-683*) in which we elaborate on the implications of these results for our findings regarding mental states and dehumanization theory (*lines 663-671*) and how they bolster accounts of factors thought to have facilitated the Nazi Holocaust (*lines 672-683*).

**Contextualizing our dataset.** Although Reviewer 4 noted that the size of our dataset (approximately 57,000 words spanning 58 months) “seems sufficiently sizable for statistical analysis and drawing certain logical inferences”, they advised us to make the sufficient size of our dataset more explicit by referencing methodological discussions of sample size for linguistic analyses. We have now done so when introducing our data in the Method section, saying “The number of text and observations in this dataset produce valid estimates using the modeling approach we employed” and providing supporting references ([Jebb et al., 2015](https://doi.org/10.3389/fpsyg.2015.00727); [McCleary et al., 1980](https://www.ojp.gov/ncjrs/virtual-library/abstracts/applied-time-series-analysis-social-sciences); *lines 224-226*).

**Justification for foregoing correction for experiment-wise error.** As per Reviewer 4’s suggestion, we now provide justification for foregoing correction for experiment-wise error for the multiple tests performed when analyzing the additional linguistic constructs in the “Analysis plan” subsection of the Method (*lines 327-331*): “Because these analyses followed a set of specified hypotheses based on the constructs’ relation to dehumanization (and more specifically, mind denial) and historical accounts of Nazi propaganda, we opted not to adjust our criterion for significance to account for experiment-wide error rate—which would constrain statistical power and may inflate risks of Type-II errors ([Bender & Lange, 2001](https://doi.org/10.1016/S0895-4356(00)00314-0); [Perneger, 1998](https://doi.org/10.1136/bmj.316.7139.1236)).”

**Confidence intervals and effect size**. As per Reviewer 4’s suggestions, we have now added confidence intervals for all results reported in the main text and Supporting Information, as well as a measure of effect size (Cohen’s *d*) for the two-sample *t*-test reported in the Supporting Information (*line 55*).

**Noting small effect sizes**. Reviewer 4 suggested we draw more attention to the magnitude of our observed effects in order to help readers appraise our conclusions. We wholeheartedly agree with this suggestion. Indeed, in our Discussion, we explicitly draw attention to this issue, noting that “many of the effects we observed were small in magnitude” (*line 695*) and discussing several possibilities for this. Moreover, we now explicitly “note that the magnitudes of several statistically significant effects were small” in the Results section as well (*line 564*).

**Discussion**. Reviewer 4 suggested that the start of the Discussion would be made stronger and clearer if explicitly tied back to hypotheses, so we have now done so (*lines 567-574*): “Dehumanization is often evoked to explain instances of mass violence. Proponents of this view claim that the denial of their victims’ humanity enables perpetrators to overcome moral inhibitions against harming conspecifics (e.g., [1,3,4]). However, empirical evidence linking dehumanization to actual instances of mass violence is lacking. Moreover, a growing body of scholars have questioned this “moral disengagement” hypothesis by pointing out that perpetrators often inflict harm precisely because they recognize their victims’ capacity for fundamentally human mental states—concluding that dehumanization is of limited relevance to mass violence (e.g., [6,16,20–22]).”

**Limitations of our data**. Reviewer 4 noted an important limitation to the present research that we did not draw attention to in our initial version of the manuscript. Namely, they pointed out that there is “Inherent uncertainty about the comprehensiveness of the sampling source.” Therefore, we now note that “although the German Propaganda Archive is impressive in the volume and diversity of material it contains, it should in no way be considered representative of propaganda produced under the Nazi regime. Therefore, we consider our results suggestive rather than conclusive and encourage future research to investigate more comprehensive corpora of Nazi propaganda“ in the “Limitations” subsection of our Discussion (*lines 690-694*).

**Concluding remarks**. We again thank the editor and reviewers for their careful consideration of our manuscript. We did our best to integrate this feedback, and would genuinely appreciate your reconsideration of what is hopefully a substantially improved paper.

Sincerely,

Alexander Landry

Doctoral Student, Organizational Behavior

Stanford University
