# Supplementary material for: Dehumanization and mass violence: A study of mental state language in Nazi propaganda (1927–1945)
Source: PLoS One. 2022 Nov 9;17(11):e0274957. doi: 10.1371/journal.pone.0274957 (PMC9645591; doi:10.1371/journal.pone.0274957)
Supplement: Attachment — Submitted filename: Response to Reviewers.docx [file pone.0274957.s004.docx]

August 22, 2022

To whom it may concern:

My colleagues and I would first like to sincerely thank the editor and four anonymous reviewers for their continued consideration of our manuscript, entitled “Dehumanization and mass violence: A study of mental state language in Nazi propaganda (1927-1945)” (Manuscript ID: PONE-D-21-38261).

We received gracious feedback from the reviewers on July 20, 2022. Although we were able to successfully incorporate many of their suggestions to improve our piece, it was brought to our attention that there were still remaining points from Reviewer 4 to address. In the following, I will respond to each remaining point raised by Reviewer 4, noting our revisions, in the order they appeared in the decision letter. We again thank all four reviewers and the action editor for the time and effort they have taken to thoughtfully consider this work.

**Restructuring Introduction.** We have now restructured the subsection of our Introduction in light of Reviewer 4’s suggestion: *Onset of the Holocaust > A note on perpetrators > Hypotheses > Additional linguistic constructs* (pp. 4-7). We agree this allows for a cleaner narrative progression. We have also relabeled the final subsection to “Exploratory analyses of additional linguistic constructs” (*line 172*).

**Limitations of our Data.** Reviewer 4 suggested we transition our comments about the limitations of our data (lines 336-344) from the Methods to Discussion section. However, we felt it important to highlight the limitations of our data *before* presenting the analyses and results, so readers can more accurately appraise and interpret said results. Indeed, in this section we explicitly “encourage readers to keep these limitations in mind when evaluating our results.” Therefore, we elected to keep this subsection in the Methods section and hope Reviewer 4 understands our perspective.

**Moving Operationalizations to Methods.** In the initial revision of this manuscript, we inadvertently provided operational—rather than purely conceptual—definitions of the additional linguistic constructs in the Introduction, as opposed to Methods section. Therefore, we agree with Reviewer 4’s recommendation that we operationalize all constructs in the Methods. We conceptually introduce each of the additional linguistic constructs in the Introduction (see “Exploratory analyses of additional linguistic constructs” subsection on pp. 5-7) before proceeding to operationally define them in the Methods (see “Psycholinguistic tools for additional linguistic constructs” subsection of pp. 9-10).

**Justifying Unit of Analysis**. Reviewer 4 suggested we incorporate our justification for analyzing the data monthly, vs. alternative possibilities, into the main text. We have now done this (*lines 256-259*).

**Discussing Results as Consistent or Inconsistent with Hypotheses.** Reviewer 4 asked us to be more explicit regarding whether the patterns in mental state terms we observed we consistent with our hypotheses or not. However, we were intentionally reluctant to do so because we considered this study as an attempt to adjudicate between two compelling accounts that have both received theoretical and empirical support—and framed the introduction as such. For instance, note that we never say *we* agreed with the moral disengagement or limited relevance perspectives. Rather, this is how we lay out the hypotheses: “If mind denial facilitates violence by undermining moral concern, we would expect a negative trend in mental state terms from 1927 to the onset of the Holocaust. Conversely, if mind denial is of limited relevance to the instigation of mass violence, we would expect no consistent trend in mental state terms preceding the Holocaust.” We feel the balanced tone we adopt in the Discussion is consistent with our framing in the Introduction.

**Fusing Subsections in Discussion.** We are thankful for Reviewer 4’s suggestion to fuse the “Speculations on increasing agency” and “Additional linguistic constructs” subsections of our Discussion in the interest of consistent organization of constructs. We have now done this (*lines 478-488*).

**Noting Additional Limitations.** Reviewer 4 pointed out that we neglected to note two additional limitations they pointed out in their initial review. Namely, the lack of reported inter-rater reliability results for data extraction and the potential for some of the statistically significant but small-sized results being spurious due to a lack of controlling for risk of experiment-wise error. We have now noted both of these limitations (*lines 557-558; 561-563*).

**Concluding remarks**. We again thank the editor and reviewers for their continued consideration of our manuscript. We did our best to integrate the remaining feedback we received, and would genuinely appreciate your reconsideration of what is hopefully an improved paper.

Sincerely,

Alexander Landry

Doctoral Student, Organizational Behavior

Stanford University
